# Effectiveness of a school-based Life Gatekeeper Training Program on suicide prevention in China: protocol for a randomized controlled trial

**DOI:** 10.1186/s13063-024-08137-2

**Published:** 2024-05-21

**Authors:** Diyang Qu, Xuan Zhang, Dongyu Liu, Bowen Liu, Dongyang Chen, Chengxi Cai, Jing An, Shekhar Saxena, Runsen Chen

**Affiliations:** 1 Vanke School of Public Health, Tsinghua University, Beijing, 100084 China; 2 Institute for Healthy China, Tsinghua University, Beijing, China; 3 Department of Global Health and Population, Harvard T. H. Chan School of Public Health, Boston, USA; 4 Beijing Huilongguan Hospital, Beijing, China; 5 Peking University Huilongguan Clinical Medical School, Beijing, China; 6 WHO Collaborating Center for Research and Training in Suicide Prevention, Beijing, China

**Keywords:** Life gatekeeper training, Suicide prevention, Children and adolescents, Implementation science, School-based prevention program

## Abstract

**Background:**

With suicide as a leading cause of death, the issue of children and adolescent suicide risks is in the spotlight today. To empower teachers in primary and secondary schools to serve as gatekeepers and to ensure the safety of children and adolescents, the systematically tailored and localized Life Gatekeeper suicide prevention program was designed for Chinese schools.

**Objective:**

With the ultimate goal of preventing child and adolescent suicide, we aim to outline a research protocol for examining outcomes of the recently created standardized school-based Life Gatekeeper program in reducing teachers’ stigma, increasing their knowledge, willingness to intervene, and perceived competence.

**Methods:**

Participants will be recruited from eligible primary and secondary schools. Cluster sampling will be used to randomly assign each school to either the intervention group or the control group. The primary outcomes are stigma against suicide, suicide literacy, perceived competence, and willingness to intervene with suicidal individuals, which will be measured using the Stigma of Suicide Scale, the Literacy of Suicide Scale, and the Willingness to Intervene Against Suicide Questionnaire, respectively. Measurements will be taken at four time points, including pre-intervention, immediately after the intervention, 6-month follow-up, and 1-year follow-up.

**Conclusions:**

The current study features innovative implementation in the real world, by using a randomized controlled trial design to examine the effectiveness of a school-based gatekeeper program among primary and secondary school teachers, following a sequence of defined and refined steps. The research will also investigate the viability of a school-based gatekeeper program for primary and secondary school teachers that could be quickly and inexpensively implemented in a large number of schools.

**Supplementary Information:**

The online version contains supplementary material available at 10.1186/s13063-024-08137-2.

## Introduction

Researchers recognize suicide as one of the most severe global public health concerns. By 2022, the global lifetime prevalence of youth active suicidal ideation and suicide attempts was 20.4 and 7.0%, respectively, according to a meta-analysis [1]. This issue has also been a matter of public concern in China. For example, a cross-sectional study found that the prevalence of suicidal ideation for primary, middle, and high school students was 30.4, 34.7, and 35.1% among 127,333 children and adolescents from several areas in China [2]. In addition, another meta-analysis study also found the prevalence of suicide attempts being 2.9% among 200,124 Chinese adolescents [3].

Suicide not only affects the long-term psychological development of children and adolescents, but it can also severely affect their family members and cause significant damage to family systems, which in turn could lead to maladaptive outcomes for children and adolescents [4, 5]. Moreover, individuals who have lost a friend to suicide often struggle with psychological distress such as depression and grief, or even suicidal ideation [6]. From a teacher’s perspective, the incident may also frequently lead teachers to develop symptoms of depression and sleep problems [7].

Recently, there has been increasing attention on the mental health of children and adolescents within the school environment, acknowledged as the “frontline of suicide prevention” [8]. Surprisingly, the mental health support available within the school system remains insufficient, which could be attributed to the ratio of 1:1360 between school psychological service providers and students in some Chinese schools [9], highlighting the concerning reality of limited access to mental health services for students. On the other hand, some at-risk students might have decreased willingness to seek professional help when struggling with mental health problems, which when combined with various effects of stigma, such as social isolation and hopelessness, may further raise their risk of suicide [10, 11]. Furthermore, the existence of some specific reasons in low-resource areas makes child and adolescent suicide more likely to be overlooked. For instance, for left-behind children, as their parents left to make a living in urban areas, their children are typically left at home with the older members of the family [12], hence these children’s ability to seek help might be hindered by the limited support from their parents. Such limitation puts an extra challenge for early identification of students at risk of suicide, hence impeding the implementation of timely interventions that could prevent the further development of suicidal risks. Therefore, there is an urgent need to establish an effective network that can increase teacher-parent-student communication to safeguard children and adolescents in low-resource areas from suicide [13].

The Gatekeeper Training (GKT) program could be a potential solution to this problem. It equips lay people with the skills to identify, assess, connect, and refer people at risk of suicide to clinicians and hospitals. This type of program could address the shortage of licensed practitioners and resources in rural areas [14–16]. For example, Question, Persuade, Refer (QPR) [17–20], Applied Suicide Intervention Skills Training [21–24], and Suicide CARE (“Careful observation,” “Active listening,” and “Risk evaluation and Expert referral” [25]) have been well-known GKTs. In addition, a systematic review of gatekeeper training for adolescent suicide prevention concluded that most GKTs had improved gatekeeper's knowledge, attitudes, self-efficacy, skills, and likelihood of intervention [26]. Overall, existing gatekeeper programs, including school-based gatekeeper training, have been found to reduce stigma, improve knowledge and self-efficacy to prevent suicide, and ultimately encourage helping behaviors among gatekeepers.

However, to the best of our knowledge, there remains a need to optimize and enrich a systematic, localized, standardized GKT program specifically designed for school teachers in China. Thus, the Life Gatekeeper Training Program has been developed to decrease suicidal behaviors among Chinese children and adolescents through teachers’ interventions. This program aims to empower school teachers to act as life gatekeepers for students experiencing psychological distress, to increase teachers’ knowledge of suicide, reduce their stigma towards suicide, and improve their skills, willingness to intervene, and perceived competence. These factors ultimately contribute to their capability as gatekeepers, which in turn could prevent child and adolescent suicide. Informed by the Theory of Planned Behavior (TPB) that shapes the program’s conceptualization, it asserts that actual gatekeeper behaviors can be anticipated by intervention intention, which is influenced by attitude, subjective norms, and perceived behavioral control [27].

Additionally, considering the high demand for communication with parents and following the most important findings from a previous Delphi study [28], this program includes a special section on teacher-parent-student communication concerning the suicidal risks of the student. Moreover, handouts for parents include a pamphlet that aims to increase the parents’ suicide literacy and hence reduce their stigma towards suicide, and an information sheet outlining mental health service resources and crisis hotlines. Overall, the training program aims to establish an effective school-family-professional institution multi-level network that protects children and adolescents from building up suicidal risks.

Meanwhile, implementing such training in the real-world setting remains a practical barrier due to the lack of human resources and expertises to spread across a large number of schools in rural areas. A strategy known as “Train-the-Trainer (TTT)” model has emerged as a potential solution, as it has been extensively employed in studies involving the distribution and implementation of novel interventions, similar to the Applied Suicide Intervention Skills Training [23]. The TTT model involves instructing individuals within a particular field on a specific subject, providing them with training and guidance on how to subsequently train, oversee, and supervise others using this approach [29]. This method is considered more viable and cost-effective for quick dissemination, although it also raises concerns regarding standardization and potential inconsistencies in training content by different trainers. Therefore, the standardized curriculum and video-based training were utilized to ensure consistent and high-quality training across different facilities and locations, as our pilot study has already been proven to be at least as effective as the traditional format of training [30].

Taken together, a systematically localized GKT was created for children and adolescents. The aim of this study was to ascertain the effectiveness of a well-established school-based Life Gatekeeper Training Program tailored for school teachers in low-resource areas. The specific focus was on enhancing teachers’ knowledge, beliefs, skills, willingness to intervene, and perceived competence in suicide prevention and was evaluated through a randomized controlled trial.

## Methods

### Trial design

**Fig. 1:**
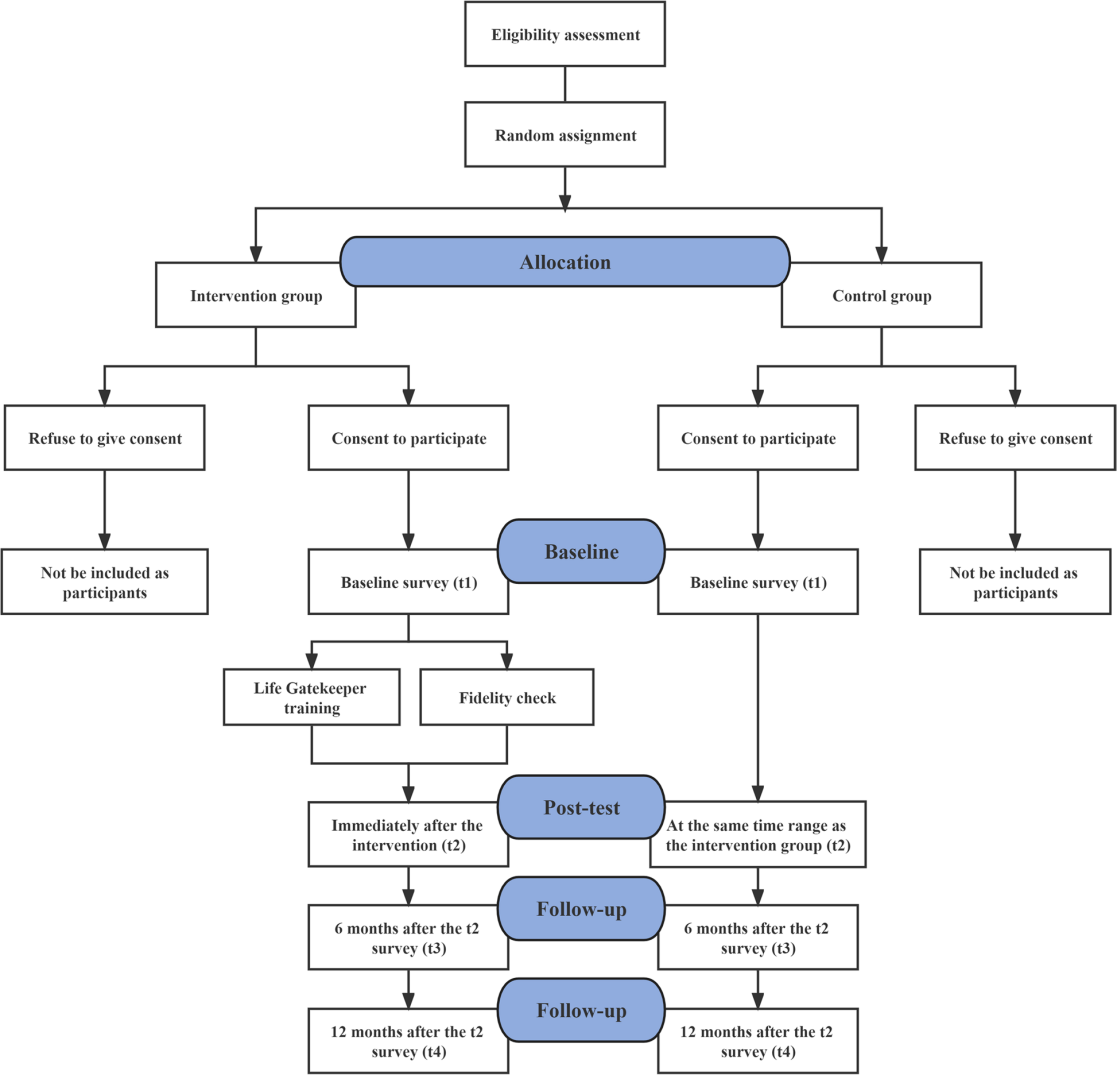
Participant flowchart

The participant flow and research design are depicted in Fig. 1. This trial will be a two-arm, non-blinded randomized controlled trial (RCT) with two parallel groups: a wait-list control group and a Life Gatekeeper intervention group. All qualified teachers in the same school will be assigned to either the intervention group or the control group through cluster sampling. The day before intervention group training, participants will be required to take a baseline survey (*t*_1_). The same day the training is finished, the post-test survey (*t*_2_: immediately following the intervention) will be carried out. Six months (*t*_3_) and twelve months (*t*_4_) after the *t*_2_ survey, respectively, follow-up questionnaires will be completed. The survey forms will be used for all polls, and the same time frame applies to the control group. The Standard Protocol Items: Recommendations for Interventional Trials (SPIRIT) standards were followed in the preparation of this manuscript (Supplementary Material 1).

### Participants and setting

Teachers in primary and secondary schools will be the target of this study. Teachers who are employed full-time will be included as participants. Teachers who have previously taken part in any systematic gatekeeper program training will be excluded from consideration. Participant recruitment was led by the primary investigator RC in cooperation with the local education bureau. DQ was responsible for field work of participant recruitment, with recruitment team members including BL, DL, XZ, and DC. The study will be conducted in Yunfu City, in the Guangdong province of China.

### Procedure

Each school will be randomly assigned to either the intervention group or the control group using cluster sampling. The researchers will inform participants of their group allocation. Participants can learn about the Life Gatekeeper training study by scanning a given QR code and then reading a detailed explanation of its purposes and procedures. All participants will be asked to provide informed consent, and the data gathered will be kept confidential. Teachers will be trained using a method referred to as the TTT model. In the Life Gatekeeper Training Program, professional instructors first conduct primary remote training for the intervention group’s core teachers. Over the week following the primary training, the intervention group’s core teachers conduct secondary in-person training for all head teachers and teaching staff from their own schools. To ensure the intervention is delivered as specified in the intervention protocol or manual, professional instructors and core teachers will complete the fidelity checklist while carrying out each training [31]. Each participant will complete the baseline survey (*t*_1_) one day before the training of the intervention group, and the post-test survey (*t*_2_) immediately after the training. These participants will also receive follow-up surveys at 6 months (*t*_3_) and 12 months (*t*_4_) after *t*_2_. Surveys at time points 1, 2, 3, and 4 will be carried out synchronously for both the control and intervention groups. Participants in the control group, including core teachers, head of class teachers, and teaching staff, will be offered the Life Gatekeeper Training Program after the 12-month follow-up. Training certificates issued by the research team will be used to promote participant retention. Data collection training will be conducted for the team members before the study, and core teachers will also be trained to cooperate with the team members in collecting data in their respective schools. If any participant discontinues participation between baseline and the 12-month follow-up, no additional data will be collected.

### Intervention program

The following steps were used to create the first video-delivered school-based gatekeeper program in China. First, the Delphi method was utilized, engaging a group of experts who independently rated their agreement with the proposed content for the program in a multi-stage, iterative manner. Experts were individually invited through email if they were specialists with relevant suicide prevention or intervention experience. After recruitment, two rounds of structured Delphi surveys were used to collect expert feedback on the following aspects: the importance of the training content, the feasibility of the training method, the achievement of the training objectives, and the appropriateness of the training materials. In the first round, 157 items were reviewed by 34 panel members, and in the second round, 55 items were reviewed by 31 panel members. Items that were endorsed by 80% or more of the panel members were adopted immediately. Overall, 201 statements were endorsed to be included in the Life Gatekeeper Training Program [28]. Then, two single-arm sequential pilot studies with 57 teachers fromtwo schools were conducted and evaluated for the program’s effectiveness, employing in-person training and then hybrid delivery strategies (i.e., remote and in-person training)  followed by the TTT approach. Both delivery methods have been proven effective, with the TTT version, in particular, showing advantages in practical implementation [30]. Finally, we modified the program and generated the final version of the Life Gatekeeper Training Program based on the findings of the pilot studies. The first school-based gatekeeper program for primary and secondary school teachers has been established in its final form.

The Life Gatekeeper Training Program consists of two main packages. The first package comprises eight sessions of video-recorded training (Table 1), including psychoeducational content, animated films, role-playing, and group discussions, with the training concluding with online group supervision by crisis intervention specialists. For instance, when it is time for the group discussion, there will be a prompt in the video indicating, “It’s time for the group discussion.” A timer will be displayed on the screen, and the instructors or core teachers only need to organize the discussion activity. In addition, the following topics are covered within the program (1) the severity of suicide among children and adolescents, and the common feelings of suicidal persons, (2) establishing a correct understanding of suicide, (3a) risk factors associated with suicide, (3b) identifying the warning signs of suicide, (4) the correct way to communicate suicide risk, (5) assessing suicide risk, (6) making a safety plan, (7) teachers communicating with parents about their kids’ suicide risk, psychoeducating parents on how to express support to their kids, and determining subsequent procedures with parents, and (8) any potential barrier that might stop teachers from providing help, and how to overcome it into actual gatekeeping behaviors.

**Table 1:** Contents of the Life Gatekeeper Training Program

Section	Theme	Methods of delivery	Length of training
1	The severity of suicide among children and adolescents, and the common feelings of suicidal persons	Video viewing; group discussion	21 min + 07 sec
2	Establishing a correct understanding of suicide	Video viewing; group discussion	22 min + 35 sec
3	Risk factors associated with suicide, identifying the warning signs of suicide	Video viewing; group discussion	22 min + 12 sec
4	The correct way to communicate suicide risk	Video viewing; group discussion; role play	66 min + 48 sec
5	Assessing suicide risk	Video viewing; role play	47 min + 27 sec
6	Making a safety plan	Video viewing; role play	78 min + 58 sec
7	Teachers communicating with parents about their kids’ suicide risk, psychoeducating parents on how to express support to their kids, and determining subsequent procedures with parents	Video viewing; role play	43 min + 13 sec
8	Any potential barrier that might stop teachers from providing help, and how to overcome it into actual gatekeeping behaviors	Video viewing; group discussion	15 min + 40 sec

*min* minutes, *sec* seconds

All participants will also receive another package, including a training manual, an appendix, a pamphlet for parents, a pamphlet for children and adolescents, and an information sheet summarizing mental health service resources and crisis hotline numbers, in addition to these recordings. The training manual and appendix will include everything that will be covered in training. The pamphlet for parents can be used to educate parents of at-risk students about child and adolescent suicide and how to seek help. During a crisis, students can use the pamphlet for children and adolescents to find support for themselves. Meanwhile, teachers can provide at-risk students and their parents with an information sheet on mental health services and crisis hotline numbers, so that they can refer students to receive professional assistance as needed. These materials will be printed out in advance and utilized for role-plays, group discussions, and note-taking during the training. All required materials were stored in a password-protected cloud storage platform to make the materials easily accessible.

The intervention is delivered in a single day, with a break in the middle. Only members in the intervention group will have access to all resources, including video clips, as they are only available to individuals who consent to participate. Nevertheless, teachers are free to hand out the pamphlet for parents, the pamphlet for children and adolescents, and the summary sheet later in their work with at-risk students. The fidelity checklist was employed to ensure that the trainers followed each step correctly, and to guarantee consistent audiovisual delivery and adherence to the procedure.

### Outcomes

The outcome measures are summarized in Table 2. All participants will be assessed for the primary and secondary outcomes, while only participants in the intervention group will be assessed for the process evaluation outcomes at the *t*_2_ questionnaire.

**Table 2:** O﻿utcome measures for the randomized controlled trial of the Life Gatekeeper Training Program

Measurements	Aim	t_1_	t_2_	t_3_	t_4_
**Primary outcomes**					
Short form of the stigma of suicide scale – stigma subscale	Stigmatized attitudes against suicide	✓	✓	✓	✓
Short form of the literacy of suicide scale	Suicide literacy	✓	✓	✓	✓
The willingness to intervene against suicide questionnaire – perceived behavioral control subscale	Perceived competence to intervene	✓	✓	✓	✓
The willingness to intervene against suicide questionnaire – willingness to intervene subscale	Willingness to in intervene	✓	✓	✓	✓
**Secondary outcomes**					
Gatekeeper behaviors	Behaviors as gatekeeper after intervention			✓^*^	✓
**Process evaluation**					
Perceived intervention quality	General satisfaction, skills acquired, knowledge gained, willingness to recommend the intervention		✓		
Fidelity Checklist	Objective quality of delivering of the intervention		✓		

*Note*. The ^*^ represents a measure used exclusively for the intervention group

### Primary outcomes

#### Stigma of suicide scale

The Chinese version of the short form of the Stigma of Suicide Scale (SOSS) will be used to measure participants’ stigmatized attitudes against suicidal individuals [32]. The SOSS has three subscales; the current study will utilize the stigma subscale to measure participants’ aversive attitudes toward suicidal individuals [32]. The original stigma subscale of the SOSS has eight items, while the Chinese version included five items after local adaptation based on psychometric tests conducted with Chinese participants [33]. Participants will indicate their attitudes toward each item by using a 5-point Likert scale from 1 (strongly disagree) to 5 (strongly agree). The Chinese short form SOSS has been shown to have good psychometric properties as a measurement capturing the stigma of suicide in the Chinese population [33]. The total score is the sum of all items, with higher scores indicating more severe stigmatized attitudes.

#### Literacy of suicide scale

The Chinese version of the short form Literacy of Suicide Scale (LOSS) will be used to measure participants’ suicide literacy [33]. The LOSS is a 11-item scale covering a series of statements about suicidal behaviors and suicidal individuals. The LOSS measures one’s suicide literacy regarding the nature, symptoms, and help-seeking methods of suicide by having participants indicate whether they think a statement is true [34]. Participants will indicate their attitudes for each item by choosing from True, False, or Not sure, while a correct answer will be awarded 1 point. The Chinese short form LOSS has been shown to have good psychometric properties as a measurement capturing suicide literacy in the Chinese population [33]. The total score is the sum of correct items, with higher scores indicating greater literacy of suicide.

#### Perceived competence, and willingness to intervene against suicide questionnaire

The Chinese version of the Willingness to Intervene Against Suicide Questionnaire (WIS), will be used to measure participants’ perceived competence and willingness to intervene with students with suicidal risk [35]. The WIS, developed through three studies involving American college students, consists of four subscales. The current study will use the perceived behavioral control subscale to measure participants’ perceived competence to intervene and use the willingness to intervene subscale to measure participants’ willingness to intervene with students with suicidal risks. The perceived behavioral control subscale and the willingness to intervene subscale have 20 and 22 items respectively, capturing a range of methods of intervening with students with suicidal risks. Participants will use a 5-point Likert scale to indicate whether they feel confident or are willing to intervene with a suicidal student through different methods in the perceived behavioral control subscale and the willingness to intervene subscale, respectively. The WIS showed good psychometric properties as a measurement capturing one’s perceived competence and willingness to intervene with a student with suicidal risk [36]. The Chinese version was translated and reviewed by two researchers with previous experience in suicide research who are fluent in both Chinese and English. The total score is the sum of each item for both subscales, with higher scores indicating greater perceived competence and willingness to intervene.

### Secondary outcome

#### Gatekeeper behaviors

The study will also measure the gatekeeper behaviors of participants in both the intervention and control groups at 12 months post-intervention. To measure short-term behavioral changes, the intervention group will also report behaviors at the 6-month mark [37, 38]. This measurement will be conducted using four individual questions that have been adjusted from previous studies to enable a rigorous comparison, taking into account the lagging nature of the observation period for behavior changes. Following questions will be asked (1) During the 6 months/12 months after the training, have you identified any students who may be at risk of suicide (or are experiencing psychological distress)? (2) Among these students, how many have you discussed potential suicide risks (or their psychological distress) with? (3) Among these students, how many have you discussed their suicide risk (or their psychological distress) with their parents? (4) Among these students, how many have you referred to mental health professionals or specialist clinics? Percentage scores will be calculated for each intervention method by comparing the number of students they intervened with to the number of students they identified. These scores will range between 0 and 100%, with a higher percentage indicating a higher frequency of gatekeeper behaviors.

### Sample size calculation

Since the current study is designed to be a clustered randomization study, the sample size calculation is based on the number of clusters and the between-cluster coefficient of variation. Sample size calculation was conducted through the cpa.count function in “clusterPower” package in R. The cpa.count is a tool for computing the power, number of clusters needed, number of subjects per cluster needed, or other key parameters for a simple parallel cluster randomized trial with a count outcome [39]. The between-cluster coefficient of variation for the current study is identified as 0.01 based on the reference number defined at “carriage outcomes at 12 months in rural area” [40]. Since we aimed to capture small changes in the outcomes measure, a detectable effect size was set as *r* = 0.1 for power analysis. The number of schools fulfilling inclusion criteria in the local area was 84, which means each group will involve 42 clusters. With a power of 0.85 and alpha=0.05, the mean number of participants calculated for each cluster (school) is 30. A final sample in each group should thus achieve 1260. To account for a withdraw rate of 30%, we will recruit 1800 participants for each group. 

### Randomization

The participants will be assigned to the control group or the intervention group through clustered randomization methods by schools. The names of the enrolled schools will be listed in a column in Microsoft Excel. A list of random numbers will be generated in the adjusting column by the RANDOM function in Microsoft Excel, in which each school will have a randomly generated number in its corresponding row. The schools will then be ordered by the generated random number from large to small, in which 42 schools with larger corresponding numbers will be assigned to intervention group, and the other 42 schools with smaller corresponding numbers will be assigned to control group. The allocation process will be managed by two research team members PC and DL. All participants within the same school will be assigned to one of the groups altogether, no stratification will be applied. After group allocation, DQ, XZ, DL, BL, and DC will be responsible for enrolling schools and core teachers into the intervention. After completing the core teachers’ training, core teachers from each individual school will work with the aforementioned researchers to enroll other participants from their respective schools to the intervention.

### Blinding

Although participants may be able to infer that they received suicide prevention training based on the intervention materials, they will not be blinded. However, the outcome assessors and statisticians performing the statistical analyses will be blinded to the participants’ allocation.

### Statistical analysis

All statistical analysis will be conducted through R 4.1.0 Analyses. The primary outcomes will be conducted through generalized estimating equations modeling while controlling for all the demographic information [41]. The generalized model will be conducted to analyze the within-group and between-group differences at each time point for all primary outcomes in head-of-class teachers and education staffs. The same model will be applied to core teachers as a supplementary analysis. A 2-sided significance level will be set, and false discovery rate corrections will be applied to account for the risk of false positive generated by multiple comparisons. Descriptive statistics will be conducted for demographic information and process evaluation questions. Analyses will be conducted after the completion of post-intervention, 6-month follow-up, and 12-month follow-up data collection. Since there are no anticipated problems that are detrimental to the participant, no stopping guidelines are set.

### Oversight and monitoring

The investigator team includes DQ, XZ, DL, BL, DC, CC, JA, SS, CY, YS, CZ, YH, SW, PC, DR, ZL, JY, NY, YD and RC, and the trial steering group includes DQ, XZ, DL, BL, DC, CC, JA, SS, and RC. The steering committee is responsible for trial protocol designing, statistical analysis plan, data collection plan, ethical conduct, trial conduct, and contractual obligations. The trial will be sponsored by the Vanke School of Public Health, Tsinghua University, with the primary investigator, RC, receiving the funding. RC will be responsible for managing the research staff, overseeing the budget, obtaining indemnity insurance, and managing legal liability. The Vanke School of Public Health’s Academic Committee will oversee the trial. The investigator team will hold fortnightly meetings to report the progress of the intervention to the school.

### Data management and confidentiality

RC and DQ will oversee the data collection and management procedure, which will involve electronic data entry. Participants in both groups will respond to the questionnaires via online platform at all data collection time points. The data will be directly downloaded as an Excel file with pre-determined scores and orders for each question and response. Data file from each school will be merged by the analysts to create the data frame for analysis. Range checks for data values will be conducted by analysts before statistical analysis.

The current study will not collect any personal information. Participants will be assigned individual trial identification numbers for the purpose of anonymization. Organizers will record and store all data collected in the study in an electronic database on a secure server build. Only the investigators, operators, and team members have access to a secured and already been anonymized database upon their request to the principal investigator.

### Research ethics and approval

Ethical approval was granted by the Institution Review Board of Tsinghua University. Project No: 20220128. The current study was pre-registered with Chinese Clinical Trial Registry, with a registration number of ChiCTR2200066142. All participants will be debriefed about the procedure of the study and explained their right to withdraw at any time during the trial. We will advise the participants to leave the venue if they feel distressed at any point of the intervention and will refer participants to professional help if deemed necessary.

### Withdraw from the trial

Participants could withdraw from the trial for any reason at any moment. Moreover, we will assemble a team of psychotherapists and psychiatrists dedicated to crisis intervention. In instances where children and adolescents face an imminent risk of suicide, schools and teachers can contact the team. Our professionals will provide advice and support online, assisting the school teachers who require guidance through the process. After the crisis is resolved, the student will be referred to appropriate services for further treatment.

### Governance and oversight of the trial

We will establish a trial management group (TMG, consisting of the principal investigator RC, the co-principal investigators, and program operators) that will oversee the entire process of the research, including recruitment, assessments, ethical issues, adverse events, and data management, following the practices established in previous studies. The core group will meet once a week during the active phases of research, and three times during the waiting phases.

In case of any changes to the protocol, the trial management group will have an internal discussion, notify other team members, and update in the clinical trial registry.

## Discussion

### Strengths of the study

According to our hypothesis, the Life Gatekeeper Training Program for teachers will lessen the suicide stigma while enhancing their knowledge, skills, and willingness to intervene as gatekeepers, leading to increased actual gatekeeper conducts.

To the best of our knowledge, this is the first RCT study to examine a localized gatekeeper program’s effectiveness, including video-delivered components for primary and secondary school teachers. The program is practical due to its strong emphasis on skill acquisition and practice. It is also instantly accessible to participants as all video clips and workbooks can be retrieved through an online platform anytime and anywhere. Along with advancing the field of suicide prevention and intervention study in China, the proposed program will produce valuable and meaningful evidence-based practice and policy benefits for pertinent stakeholders. Ultimately, it could potentially establish a multi-level suicide prevention system that consists of schools, families, and professional psychological institutions, thereby better safeguarding children and adolescents from suicide.

### Dissemination of the findings

Publications in peer-reviewed journals will be used to disseminate the major findings of this study. If the Life Gatekeeper  Training Program is proven to be effective, it will be disseminated to a vast number of teachers from schools in China. The promising result is that it will enable suicide prevention and enhancing children’s and adolescents’ mental health at an affordable cost, particularly in Chinese rural or low-resource areas. The Life Gatekeeper Training Program requires less input from mental health practitioners because all content is pre-recorded. In areas with limited resources, namely nearly one third of the Chinese areas, it can be implemented at a minimal cost. As a result, this Life Gatekeeper Training Program has invaluable potential to serve as a valuable and practical tool for suicide prevention in schools.

## Conclusion

This is the first study to examine, using a RCT methodology, the effectiveness of the Life Gatekeeper Training Program for Chinese school teachers to enhance their literacy, skills, and actual gatekeeper behaviors to prevent suicide among students. This study will examine the possibilities for a video-delivered school-based Life Gatekeeper Training Program that could be offered at a small cost to a large number of teachers who may then become the gatekeepers for their students.

## Trial status

This is the final study protocol as of December 2022 (Version 2.0). Participant recruitment commenced in December 2022 and terminated by March 2023. The timeline discrepancy, attributed to COVID-19, caused delays in initiating recruitment at certain schools. As of September 2023, the participants have completed data collection for the second follow-up, and we anticipate the final follow-up to end by March 2024.

### Supplementary Information

**Supplementary Material 1.**

## Data Availability

The datasets analyzed during the current study and statistical code are available from the corresponding author on reasonable request.

## Abbreviations

TTT: Train-the-Trainer
QPR: Question, Persuade, Refer
ASIST: Applied Suicide Intervention Skills Training
CARE: “Careful observation,” “Active listening,” and “Risk evaluation and Expert referral”
RCT: Randomized controlled trial
LOSS: Literacy of Suicide Scale
SOSS: Stigma of Suicide Scale
SPIRIT: The Standard Protocol Items: Recommendations for Interventional Trials
TPB: Theory of Planned Behavior
WIS: Willingness to Intervene Against Suicide Questionnaire
GKT: Gatekeeper Training
