# Correction: Effectiveness of a school-based Life Gatekeeper Training Program on suicide prevention in China: protocol for a randomized controlled trial

**DOI:** 10.1186/s13063-024-08322-3

**Published:** 2024-07-11

**Authors:** Diyang Qu, Xuan Zhang, Dongyu Liu, Bowen Liu, Dongyang Chen, Chengxi Cai, Jing An, Shekhar Saxena, Runsen Chen

**Affiliations:** 1 Vanke School of Public Health, Tsinghua University, Beijing, 100084 China; 2 Institute for Healthy China, Tsinghua University, Beijing, China; 3 Department of Global Health and Population, Harvard T. H. Chan School of Public Health, Boston, USA; 4 Beijing Huilongguan Hospital, Beijing, China; 5 Peking University Huilongguan Clinical Medical School, Beijing, China; 6 WHO Collaborating Center for Research and Training in Suicide Prevention, Beijing, China

**Correction: Trials 25, 335 (2024)**

**https://doi.org/10.1186/s13063-024-08137-2**

Following the publication of the original article [1], we were notified that a typo was identified in the methods section. In the description of the literacy of suicide scale, it was stated that "The LOSS is a 12-item scale covering a series of statements about suicidal behaviors and suicidal individuals." The number 12 was a typo as the LOSS actually contained 11 items. This 11-item version is a result of a removal of the item "Men are more likely to commit suicide than women" from the 12-item scale due to inconsistent reporting of gender-specific suicide rates in China, as indicated in Han et al. (2017). Therefore, the correct description should be "The LOSS is a 11-item scale covering a series of statements about suicidal behaviors and suicidal individuals."

This typo does not affect any other part of the protocol or the RCT itself, as the existing citation of Han et al. (2017) and the application of the 11-item version of LOSS are correct.

The original article was corrected.
